# Modeling maladaptive decision-making in a rat version of the Iowa Gambling Task

**DOI:** 10.1186/1471-2202-12-S1-P294

**Published:** 2011-07-18

**Authors:** Vincent Valton, Alain Marchand, Francoise Dellu-Hagedorn, Peggy Seriès

**Affiliations:** 1 Institute for Adaptive and Neural Computation, The University of Edinburgh, Edinburgh, EH8 9AB, UK; 2 CNRS UMR 5287, Université de Bordeaux, 146 Rue Léo Saignat, 33076 Bordeaux Cedex, France

## 

Deficits in decision-making have been repeatedly observed in various psychiatric disorders (i.e. ADHD, Pathological Gambling, Mania, OCD and Substance Abuse) as well as in frontal lobe patients. Such decision-making deficits are often assessed using the Iowa Gambling task (IGT) [1]. The IGT represents a realistic decision-making task where subjects are asked to choose between targets associated with rewards and penalties of varying likelihood and amplitude. Previous studies have shown that when healthy participants take the IGT, around a third of these perform poorly, similar to psychiatric patients [1].

Recently, these behavioral findings were successfully translated to animal research in a rodent version of the IGT, the Rat Gambling Task (RGT). In common with human studies, it was found that a third of a healthy population of rats exhibited poor decision-making performances [2]. The rats were tested in other tasks aiming at characterizing behavioral traits such as impulsivity, sensitivity to reward, cognitive inflexibility and risk seeking. Poor decision makers were always characterized by high scores for a combination of these behavioral traits.

Here we use a model of learning and decision-making in the RGT to answer the following questions: (1) how do the behavioral traits described above influence learning; (2) how is this manifested in terms of their decision-making performance?

In order to model the learning and decision process of the RGT, we used a TD-learning algorithm [3]. The model agent experiences the environment by learning the values of rewards and penalties for each state using trial and error sampling. As the agent gets a more accurate representation of the environment, it takes more appropriate decisions, using a ‘softmax’ action selection process. The RGT is modeled as a Markov decision process and we extended the classical TD-learning algorithm by incorporating risk seeking [4], reward sensitivity and cognitive inflexibility. These behavioral traits were implemented independently and influence either the learning rate or the perception of rewards by the agent. The parameters of the model were extracted for each rat by fitting their performance to the model.

We found that the model could account for the performances of good and poor subpopulations of decision makers. Additionally, the parameters defining the behavioral traits extracted from the model correlated significantly with those measured experimentally for the poor and good decision makers’ subgroups. The model was also able to predict the inflexibility of poor decision makers during reversal conditions.

Our work supports the hypothesis that it is a combination of high scores for risk seeking, sensitivity to reward and cognitive inflexibility that lead to poor decision-making performances. According to the model, behavioral traits affect the learning process of the subjects by altering the estimated value of the received rewards and reducing their ability to reverse their initial estimations. This results in an incorrect perception of the environment, leading to an optimal decision-making according to their world representation but aberrant according to the real outcome of the task.
